# Genetic predisposition and induced pro-inflammatory/pro-oxidative status may play a role in increased atherothrombotic events in nilotinib treated chronic myeloid leukemia patients

**DOI:** 10.18632/oncotarget.11100

**Published:** 2016-08-05

**Authors:** Monica Bocchia, Sara Galimberti, Lara Aprile, Anna Sicuranza, Antonella Gozzini, Francesca Santilli, Elisabetta Abruzzese, Claudia Baratè, Barbara Scappini, Giulia Fontanelli, Monika Malgorzata Trawinska, Marzia Defina, Alessandro Gozzetti, Alberto Bosi, Mario Petrini, Luca Puccetti

**Affiliations:** ^1^ Department of Hematology, University of Siena, Azienda Ospedaliera Universitaria Senese, Siena, Italy; ^2^ Department of Clinical and Experimental Medicine, U.O. Hematology, University of Pisa, Pisa, Italy; ^3^ Functional Unit of Hematology, University of Florence, Florence, Italy; ^4^ Department of Medicine and Aging, University of Chieti, Chieti, Italy; ^5^ Hematology Unit, S. Eugenio Hospital, Tor Vergata University, Rome, Italy

**Keywords:** PAOD, TKI, CML, inflammation, atherothrombotic risk

## Abstract

Several reports described an increased risk of cardiovascular (CV) events, mainly atherothrombotic, in Chronic Myeloid Leukemia (CML) patients receiving nilotinib. However, the underlying mechanism remains elusive. The objective of the current cross-sectional retrospective study is to address a potential correlation between Tyrosine Kinase Inhibitors (TKIs) treatment and CV events. One hundred and 10 chronic phase CML patients in complete cytogenetic response during nilotinib or imatinib, were screened for CV events and evaluated for: traditional CV risk factors, pro/anti-inflammatory biochemical parameters and detrimental ORL1 gene polymorphisms (encoding for altered oxidized LDL receptor-1). Multivariate analysis of the whole cohort showed that the cluster of co-existing nilotinib treatment, dyslipidaemia and G allele of LOX-1 polymorphism was the only significant finding associated with CV events. Furthermore, multivariate analysis according to TKI treatment confirmed IVS4-14 G/G LOX-1 polymorphism as the strongest predictive factor for a higher incidence of CV events in nilotinib patients. Biochemical assessment showed an unbalanced pro-inflammatory cytokines network in nilotinib vs imatinib patients. Surprisingly, pre-existing traditional CV risk factors were not always predictive of CV events. We believe that in nilotinib patients an induced “inflammatory/oxidative status”, together with a genetic pro-atherothrombotic predisposition, may favour the increased incidence of CV events. Prospective studies focused on this issue are ongoing.

## INTRODUCTION

The great improvement in survival expectancy in Chronic Phase (CP) Chronic Myeloid Leukemia (CML) patients chronically treated with Tyrosine Kinase Inhibitors (TKIs) has prompted the clinical issue of long-term drug toxicity, safety and quality of life [1–3]. While imatinib has been generally well tolerated with an adverse effect profile stable over the years [1], long-term side effects of second and third generation TKIs are not completely defined yet and especially cardiovascular (CV) adverse events related to their use are a matter of concern [4–6]. Several recent retrospective clinical studies reported a higher incidence of atherothrombotic events including peripheral arterial occlusive disease (PAOD) in CML patients treated with nilotinib when compared to imatinib or dasatinib; these peculiar adverse events appear to increase over time during continuous nilotinib treatment [6–13]. Preliminary experimental data suggest that nilotinib, in contrast to imatinib, may cause direct pro-atherogenic, growth-inhibitory and thus anti-angiogenic effects on vascular endothelial cells [14]. However, the pathogenic link between nilotinib and atherothrombosis has not been fully elucidated and more work is needed to better understand the effects of nilotinib in preclinical CV models. From a clinical point of view, whether atherothrombotic events occur predominantly in CML patients with pre-existing traditional CV risk factors is a matter of debate as most studies are based on retrospective data and the risk factors rate for arterial occlusive disease varied significantly [15–19]. We have also taken into consideration that, according to various reports, the percentage of patients on nilotinib treatment with pre-existing CV risk factors who experienced atherothrombotic events ranged anywhere from over 90% down to 20% [9, 16, 17, 19, 20].

Given such heterogeneity of findings and clinical experience, we studied patients receiving TKIs treatment, in order to identify if the vascular events could be associated to a genetic predisposition and/or a drug induced pro-atherothrombotic effect, independently of the presence of traditional CV risk factors.

Arterial occlusive disease is mainly caused by atherosclerosis, a multifactorial disease of the vessels involving lipid and other metabolic disturbances, thrombogenic components, cell death and inflammatory responses of the arterial wall [21]. Metabolic and pro-inflammatory factors share a relevant role in the pathogenesis of atherothrombotic events mainly *via* enhanced lipid peroxidation [22]. Lectin-like oxidized-LDL (ox-LDL) receptor-1(LOX-1), is the main receptor for the pro-atherogenic ox-LDL [22–25]; it is expressed in endothelial cells, macrophages, platelets and vascular smooth muscle cells, all relevant cellular effectors of atherogenesis and atherothrombosis mechanisms [22–25]. LOX-1 gene, named OLR1, is mapped on chromosome 12p 13.2-p12.3 and different studies demonstrated that OLR1 single nucleotide polymorphisms (SNPs) may cause susceptibility to coronary artery disease (CAD), particularly in Caucasian ancestral populations [23]. In particular, the IVS4-14 A/G polymorphism, which acts in complete linkage-disequilibrium with other five polymorphisms located in the introns 4, 5 and 3′UTR region, has been implicated in geographic disparities in CV event rate [24].

Thus, to elucidate a potential pathogenic link between TKIs treatment and mechanisms underlying PAOD or other atherothrombotic events, we investigated, in a series of CML patients treated with nilotinib and imatinib, the correlation between LOX-1 polymorphisms, the presence of classic CV risk factors and vascular events. Furthermore, during treatment with either TKIs, we studied a selected pro-atherothrombotic biochemical profile evaluating the following: 1) coagulation state by Endogenous Thrombin Potential (ETP); 2) specific circulating mediators of inflammation such as IL-10, IL-6 and TNFα; 3) high-sensitivity C-reactive protein (hs-CRP) previously proven to be involved in the atherothrombosis burden [22–27]; 4) ox-LDL as metabolic effectors of atherosclerosis predisposition [22, 23, 25]; 5) soluble CD40 ligand (sCD40L) as a marker of platelet derived-inflammation and platelet activation trigger [22, 28].

## RESULTS

### Whole patients cohort data and multivariate analysis

One hundred and ten consequent CP CML patients in complete cytogenetic response (CCyR), willing to participate in the study, were evaluated. In order to consider a homogeneous cohort, to have the least active cancer-related thrombotic risk and to minimize any cytokine unbalance due to different CML cell burden at study evaluation, we included only CCyR patients.

Clinical characteristics, risk factors distribution and incidence of CV events are summarized in Table 1. The incidence of two or more traditional CV risk factors was 56%. LOX-1 genotype frequency in the whole cohort of patients respected the Hardy-Weinberg equilibrium that is present in ancestral Caucasian origin populations [24] (Table 2). Atherothrombotic events occurred in 17/110 (15%) patients thus counting for a consistent sample size for multivariate analysis. We took in consideration the TKI treatment at the time of any CV event or, if no event occurred, at the time of study visit: 58 patients were on imatinib and 52 on nilotinib treatment.

**Table 1:** Clinical characteristics and risk factors distribution of patients during TKI treatment or at the time of cardiovascular event

	whole cohort	imatinib cohort	nilotinib cohort	*p*
N° Patients	110	58	52	0.094
M/F	66/44	31/27	35/17	0.061
Median age (years, range)	61 (29-83)	59 (33-82)	62 (29-83)	0.088
Median duration of CML (months, range)	60 (3-252)	84 (12-180)	56 (3-228)	**0.019**
Median TKI exposure (months, range)	n.a.	84 (12-180)	24 (3-84)	**0.0014**
First-line (%)	n.a.	58 (100)	29 (55)	**0.0029**
Second-third line TKI (%)	n.a.	0 (0)	23 (45)	n.a.
History of smoking (%)	34 (31)	17 (29)	17 (33)	0.126
Arterial Hypertension (%)	36 (33)	17 (29)	19 (37)	0.054
Diabetes Mellitus (%)	19 (17)	9 (16)	10* (19)	0.088
BMI (range)	26±1,4	26,1±1,4	25,9±1,3	0.091
Dyslipidaemia (%)	35 (32)	15 (26)	20 (38)	**0.047**
Familiarity (%)	58 (53)	30 (52)	28 (54)	0.099
Number of risk factor > 2	62 (56)	29 (50)	33 (63)	**0.048**
Number of risk factor ≥ 3	44 (40)	18 (31)	26 (50)	**0.041**
**Total n° of cardiovascular events (%)**	17/110 (15)	3/58 (5)	14/52 (27)	**0.00011**
First line treatment (%)	n.a.	3/58 (5)	6/29 (21)	n.a.
Second-third line (%)	n.a.	0/0 (0)	8/23 (35)	n.a.
TKI treatment time to event (months)		125, 132, 102	30 (range 18-72)	n.a.

Abbreviation: CML chronic myeloid leukemia, TKI tyrosine kinase inhibitor, BMI body mass index, n.a. not applicable *2/10 glucose intolerance

**Table 2:** Distribution of oxidized-LDL receptor-1 (LOX-1) polymorphisms (IVS4-14 A/G) in study population

	all patients	imatinib whole cohort (event cohort)	nilotinib whole cohort (event cohort)	p°
**N° Patients**	110	58 (3)	52 (14)	
**A/A LOX-1 polymorphism**	33	22 (0)	11 (0)	**0.046**
**A/G LOX-1 polymorphism**	63	34 (2)	29 (2)	0.081
**G/G LOX-1 polymorphism**	14	2 (1)	12 (12)	**0.00021**
**A/A LOX-1 polymorphism (G.F. /A.F.)**	*(0.300/0.195)^§^	(0.379/0.270)	*(0.211/0.156) **(*p* = 0.048)**	
**A/G LOX-1 polymorphism (G.F./A.F.)**	*(0.572/0.642)^§^	(0.586/0.688)	*(0.557/0.601) (*p* = 0.094)	
**G/G LOX-1 polymorphism (G.F./A.F.)**	*(0.128/0.163)^§^	(0.034/0.042)	*(0.230/0.243) **(*p* = 0.0038)**	
G/G Genotype/Events relation *		p= 0.085	***p* = 0.0094**	

Abbreviation : LOX-1 oxidized-LDL receptor-1; G.F. genotype frequency; A.F. allele frequency

* χ^2^ test of independence

§ (*p* = n.s. with respect to HapMap CEU, downloaded from http://www.hapmap.org.genotypes/ and Italian population)

° Mann-Whitney U test.

#### Whole cohort multivariate analysis

**Figure 1:**
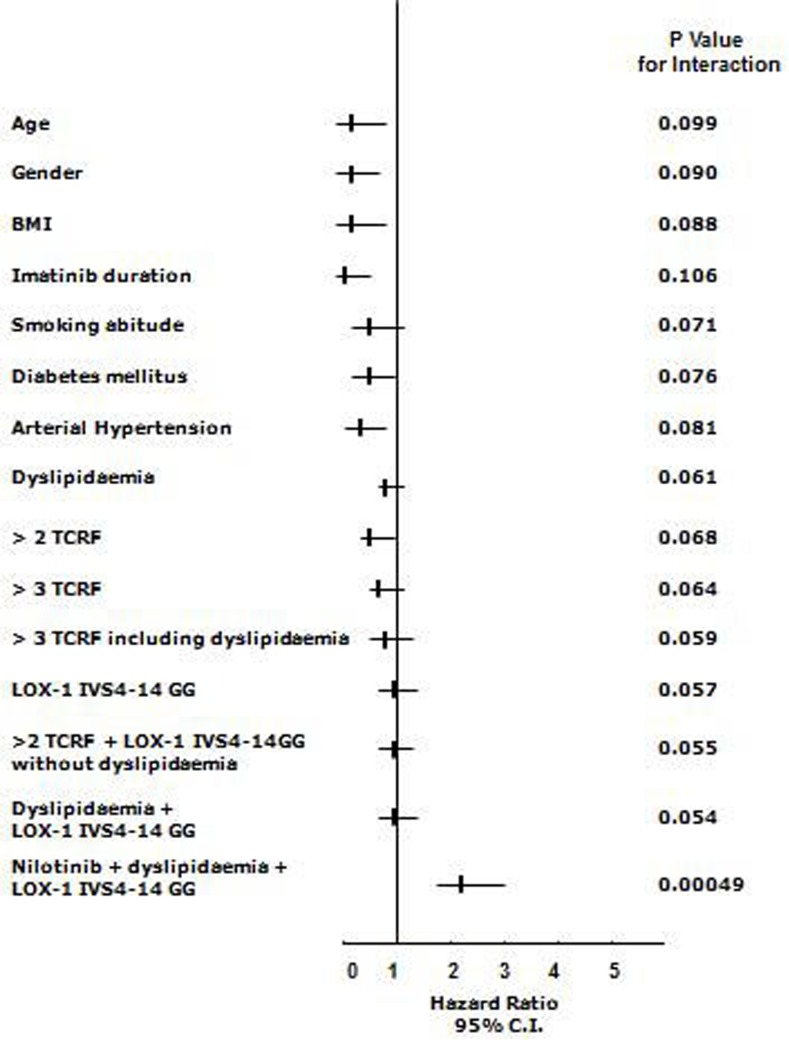
Relations among end-points and nonparametric data in the whole cohort of CML patients treated with TKIs (n= 110) Cox proportional-hazards modeling and formal test for interaction: starting from logistic regression analysis in which Y was the analyzed variable (both parametric such as age and BMI); the variables X_1_ and X_2_ were the presence or absence (1 or 0) of the LOX-1 polymorphism or other non-parametric variables (drugs, history of cardiovascular classical risk factors, here named TCRF, and X_3_ the combination). The simplified formula for calculation was: Y = β_0_+β_1_X_1_+β_2_X_2_+β_3_X_3_ and the null hypothesis was tested as H_0_: β_3_ = 0. Final data validation has been assessed with a resampling technique (exact tests) and discrimination analysis with the Hosmer–Lemeshow method [G^2^_HL_= Σ^10^_J=1_ (O_j_ – E_j_)^2^/E_j_ (1-E_j_/n_j_) ∼X^2^_8_], where nj = number of observations in the j^th^ group, Oj = Σ_1_yij = observed number of positive cases in the j^th^ group, Ej = Σpij = Expected number of positive cases in the j^th^ group. (Each reported p is obtained by this technique and considered significant if < 0.05)

The Cox Hazard model and the Hosmer-Lemeshow confirmation test, once corrected for each applicable data (age, gender, body mass index (BMI), single or clustered risk factors including smoking habit, CV medications, LOX-1 polymorphism, TKI treatment) available at the time of occurring event or at the time of clinical observation if event-free, showed that the cluster of co-existing nilotinib treatment, dyslipidaemia and the G allele of LOX-1 polymorphisms was the only significant finding associated with events (H.R. 2.19 95% C.I. 1.66-2.99, *p* < 0.001) (Figure 1). Furthermore, in patients with two or three traditional risk factors, no CV events occurred during treatment with either TKIs whether not coupled with both G allele of LOX-1 and dyslipidaemia (Figure 1).

### Patients data and multivariate analysis according to TKI treatment

Following the evidence that nilotinib treatment was a favoring factor for vascular events in the whole cohort, we further analyzed putative differences among clinical and genetic variables according to imatinib or nilotinib treatment. As reported above, at the time of any CV event or, if no event occurred, at the time of study visit, 58 patients were on imatinib and 52 on nilotinib treatment (Table 1). As expected from a “real life” unselected study population, the duration of the disease and the TKI exposure were significantly longer in the imatinib cohort when compared to the nilotinib cohort (Table 1). Additionally, 45% of nilotinib patients were on second-third line treatment. The distribution of traditional CV risk factors showed a slightly higher prevalence of dyslipidaemia in the nilotinib cohort. Similarly, having three or more CV risk factors was a more frequent observation in such group (Table 1).

An atherothrombotic event occurred in 3/58 (5%) imatinib treated patients (1 PAOD, 2 carotid occlusion > 50%), and in 14/52 (27%) patients in the nilotinib cohort (9 PAOD, 5 acute coronary syndrome) (Table 1). All three events in the imatinib group occurred during first line treatment, while 6/14 (43%) and 8/14 (57%) of events recorded in the nilotinib cohort occurred during first and second-third line treatment, respectively. Time from TKI exposure to CV event was 125, 132 and 102 months for the three patients receiving imatinib while median time of nilotinib exposure to event was 30 months (range 18-72) (Table 1).

#### LOX-1 polymorphism distribution

Considering the genotype frequency according to TKI treatment, we found a slight excess of homozygotes A/A in the imatinib group and a significant excess of homozygotes G/G in the nilotinib treated cohort (Table 2). However, it has to be noted that 23 nilotinib patients switched from a previous treatment with imatinib, thus if we consider G/G frequency according to first line therapy we found a similar distribution of the LOX-1 G/G polymorphism between the two treatment groups (imatinib first line treated cohort *n* = 81, G/G *n* = 10 = 12.4%; G.F./A.F. 0.148/0.184; nilotinib first line treated cohort *n* = 29, G/G *n* = 4 = 13.8%, G.F./A.F. 0.156/0.193, no statistically significant difference). Interestingly, the homozygotes G/G clustered in the first and second line nilotinib sub-group of patients experiencing an atherothrombotic event during treatment (Table 2).

#### Multivariate analysis

**Figure 2:**
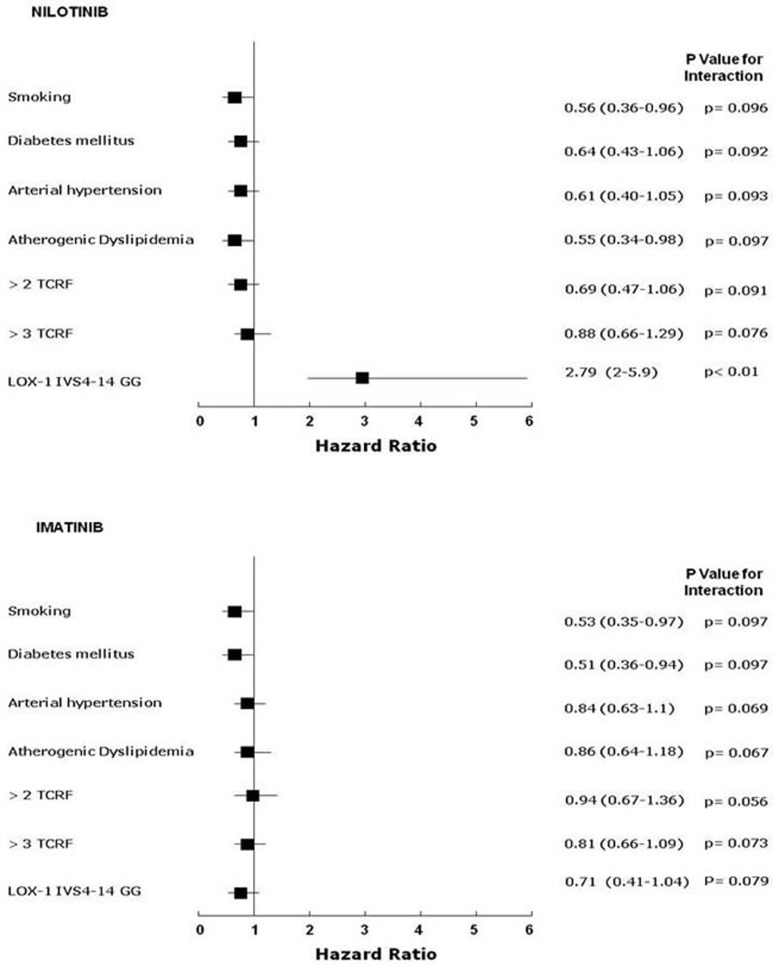
Relations among end-points and nonparametric data in imatinib treated (n=58) and nilotinib treated (n=52) CML patients Cox proportional-hazards modeling and formal test for interaction: starting from logistic regression analysis in which Y was the analyzed variable; the variable X_1_ and X_2_ were the presence or not (1 or 0) of the LOX-1 polymorphism or other non-parametric variables (history of cardiovascular classical risk factors, here named TCRF, and X_3_ the combination). The simplified formula for calculation was: Y = β_0_+β_1_X_1_+β_2_X_2_+β_3_X_3_ and the null hypothesis was tested as H_0_: β_3_ = 0. Final validation of data was assessed by a resampling technique (exact tests) and discrimination analysis by the Hosmer–Lemeshow method [G^2^_HL_= Σ^10^_J=1_ (O_j_ – E_j_)^2^/E_j_ (1-E_j_/n_j_) ∼X^28^], where nj = number of observations in the j^th^ group, Oj = Σ1yij = observed number of positive cases in the j^th^ group, Ej = Σpij = Expected number of positive cases in the j^th^ group. (Each reported p is that obtained by this technique and significant if < 0.05)

The Cox Hazard model and the Hosmer-Lemeshow confirmation test, once corrected for each applicable data (age, gender, BMI, single or clustered risk factors including smoking habit, CV medications, LOX-1 polymorphism) available at the time of event recording or at the time of clinical observation if event-free, showed that the single influencing risk factor was the G/G homozygosis for IVS4-14A/G of OLR1 in the nilotinib group (Figure 2). No significant influence on vascular event rate was detected for any single traditional CV risk factor, despite the slightly increased prevalence of dyslipidaemic subjects in the nilotinib group (Figure 2, Table 1).

### Biochemical analysis according to TKI treatment

The biochemical analysis was performed during follow-up visit in all 110 patients, yet it has to be underlined that after an atherothrombotic event some clinicians decided to switch TKI due to the adverse event. Thus biochemical assessment was performed in 65 patients on imatinib (7 of them has been previously treated with nilotinib) and in 45 patients on nilotinib treatment. Patients with a history of CV events underwent biochemical analysis after at least 6 months from the event (median time 12 months, range 6-60 months). As shown in Table 3, ox-LDL, sCD40L level and ETP, in close linear relation among each other, (Supplemental Table S1), were significantly higher in nilotinib *vs*. imatinib treated group, while IL-10 level, inversely related to ox-LDL, sCD40L and ETP (Supplemental Table S1), was significantly lower.

**Table 3:** Biochemical pro-inflammatory/pro-atherothrombotic evaluation according to TKI treatment at study visit

	imatinib whole cohort	nilotinib whole cohort	*p*	imatinib (previously treated with nilotinib)	*P* (*vs*. always treated with imatinib n. 58)
**N° Patients**	65	45		7	
**oxLDL (UI/L)**	69.9±7.1	92.2±9.9	**0.0022**	71.4±8.8	0.097
**IL6 (pg/ml)**	8.8±1.33	9.9±1.44	0.091	8.5±1.67	0.094
**IL10 (pg/ml)**	4.86±1.17	1.06±0.58	**0.00012**	4.59±1.23	0.081
**TNFα (pg/ml)**	9.6±1.8	10.8±1.93	0.094	9.9±1.9	0.088
**sCD40L (pg/ml)**	330.5±59.2	513.3±91.9	**0.0023**	336.4±55.2	0.090
**ETP (%)**	7.3±1.82	14.7±3.73	**0.0019**	7.4±1.93	0.104
**hs-CRP (mg/dl)**	1.02±0.17	1.14±0.21	0.076	1.09±0.14	0.070

Abbreviation: oxLDL oxidized-LDL, IL interleukin, TNFα tumor necrosis factor alpha, sCD40L soluble CD40 ligand, ETP Endogenous Thrombin Potential, hs-CRP high-sensibility C reactive protein.

No statistically significant differences were found in the expression of IL-6 and TNF-α between the two groups (Table 3). Interestingly, we observed no statistical difference in the cytokine profile between patients who had always been treated with imatinib and those 7 patients that were on imatinib at the time of biochemical assessment but experienced a CV event during nilotinib (Table 3).

The influence of CV risk factors and ongoing medications for CV risk control on the biochemical profile of all CML patients was also assessed. In the nilotinib cohort ox-LDL, sCD40L, ETP and IL-10 levels showed a similar pattern in patients with CV risk factors and concomitant CV drugs as well as in those without risk factors and no related treatment (Supplemental Table S1 and S2). Thus, the detrimental biochemical profile observed in nilotinib treated patients was unrelated to the underlying CV risk and remained significantly different from that observed both in the imatinib treated cohort and in the non-CML matched controls (Supplemental Table S1 and S2).

Conversely, fasting plasma glucose, lipid profile (including LDL) and hs-CRP were not significantly different between imatinib and nilotinib treated group, regardless of the presence of CV risk factors and related treatments (Table S1).

## DISCUSSION

Recently, the high occurrence of CV toxicity mainly related to atherothrombotic events during second and third generation TKIs treatment has prompted the interest and concern of clinicians caring for CML patients [13, 29]. However, the trigger of these serious adverse events has not fully clarified yet. In this preliminary cross-sectional study including a series of 110 CML patients treated with imatinib or nilotinib, we found that the cluster of co-existing nilotinib treatment, dyslipidaemia and the G allele of LOX-1 polymorphism was the only significant finding associated with atherothrombotic events.

As nilotinib appeared to be a possible causing factor for vascular events of the overall population, we further performed a multivariate analysis according to TKI treatment including age, gender, BMI, single or clustered risk factors, CV medications, LOX-1 polymorphism. The latter confirmed that the detrimental IVS4-14 G/G LOX-1 polymorphism was the strongest predictive factor for a higher incidence of atherothrombotic events in nilotinib treated patients (i.e. 14/52 (27%) *vs*. 3/58 (5%) events in nilotinib and imatinib treated patients, respectively). To our surprise, we found no clear correlation of traditional CV risk factors other than dyslipidaemia with atherothrombotic events. Indeed, we found that 12/14 (86%) of nilotinib patients with vascular events had G/G variant genotype, whereas among 26 nilotinib patients with ≥3 traditional risk factors, only 9/26 (35%) experienced an atherothrombotic event, thus implying a possible role for a different genetic profile. The distribution of LOX-1 IVS4-14 A/G polymorphisms of the whole CML population showed a close correlation with previously described Caucasian populations epidemiologic data [24]. However, with respect to treatment, the G/G distribution apparently clustered in the nilotinib treated cohort (Table 2) thus inferring a possible bias in the patients' selection. Against this hypothesis is the lack of significant between-group difference in the allelic and genetic frequency of LOX-1 when we considered patients during first line treatment (81 imatinib, 29 nilotinib) (Table 2). The apparent segregation of G/G in the nilotinib group may be due to the fact that eight G/G patients treated in first line with imatinib without evidence of any vascular event, were then switched to nilotinib (due to intolerance or no response) and only afterward experienced an event.

From a pathogenic point of view, it is known that dysfunctional LOX-1 derived from specific detrimental SNPs in the OLR-1 gene, such as the IVS4-14 A/G evaluated in our study, strongly exerts pro-atherothrombotic effects *via* disturbances of ox-LDL metabolism [23]. Indeed ox-LDL is the most active pro-atherogenic and pro-thrombotic form of lipoproteins and the process of lipid peroxidation is induced mainly by inflammatory stimuli [22, 30–33]. Therefore, in this study we have also performed a cross-sectional analysis of a selected pro-inflammatory/pro-oxidative biochemical profile during TKIs treatment. Firstly, we provided biochemical evidence of a pro-atherothrombotic profile in patients on nilotinib treatment, as highlighted by the increased sCD40L levels, and a concurrent (and correlated) enhancement of thrombin generation as compared to imatinib treated patients and matched controls. Indeed sCD40L increase was significantly associated with enhanced lipid peroxidation, as reflected by higher ox-LDL levels, suggesting that lipid peroxidation can be a relevant molecular link between inflammation and thrombosis [33]. Secondly, systemic inflammation also appeared to be enhanced, as reflected by higher hs-CRP levels in nilotinib treated subjects, although not reaching a statistical between-group significance. The evidence of an underlying pro-inflammatory state was also documented by the reduction of the anti-inflammatory cytokine IL-10 in the nilotinib cohort. In fact, the reduction of IL-10 has been described as a pro-atherogenic inflammatory condition [34] whereas hs-CRP is not a universal predictor of CV risk [35] and its levels are strictly related to IL-6 rather than other cytokines production [36]. Our data also showed a significant increase of ox-LDL levels in the nilotinib cohort of patients; this may not be due to higher circulating LDL levels but to an increased pro-oxidative, inflammatory- and genetically-driven state [37]. The finding of an increased ox-LDL level is of interest because it is independently associated with atherothrombotic events, regardless of classical parameters of lipid profile. In the general population, ox-LDL levels improved the reclassification capacity of Framingham-derived CAD risk functions [31, 32], however in our experimental model we could not estimate CV risk with this or other specific algorithms [38, 39] due to the lack of baseline data. Further evidence from specifically-designed prospective studies are needed to better clarify the involvement of traditional risk factors clustering in the development of atherothrombotic events during TKIs. An additional suggestion emerges from our study: in nilotinib patients with ongoing treatment for CV risk factors there was no apparent positive effect of CV and anti-atherothrombotic drugs on the altered ox-LDL, sCD40L, ETP and IL-10 levels.

Increase of IL-10 levels may induce an anti-atherogenic profile of LOX-1 in terms of reduction of ox-LDL uptake and minor vascular damage [40]. Thus, we have hypothesized that the pro-atherothrombotic risk during nilotinib could arise from the combination of increased lipid peroxidation due to detrimental LOX-1 polymorphism and an imbalance in cytokine-driven inflammation, mainly exerted by the strong reduction of IL-10 levels [40]. Furthermore, the activation of LOX-1 by oxLDL induces up-regulation of monocyte chemotactic factor (MCP)-1, intercellular adhesion molecule (ICAM)-1 and vascular cell adhesion molecule (VCAM)-1 [25, 41]. In this regard, Valent et al. suggest that other mechanisms potentially are involved in vascular disease during TKIs treatment [14]. They demonstrated that high levels of VCAM-1 molecules are expressed in vascular cells in nilotinib treated mice, providing a possible nilotinib “off target” effect [14].

If confirmed, this direct effect could be involved in the acceleration of plaque formation and/or destabilization and could synergize with the endothelial dysfunction induced by the altered LOX-1 activity and inflammatory state [25, 41].

## CONCLUSIONS

The combination of genetic and biochemical data here reported may provide a possible clue to unravel the underlying mechanism of atherosclerotic vascular events occurring in some nilotinib treated patients. To further confirm our pathogenic hypothesis we started a large multicenter prospective study in CP CML patients treated with any of the first line approved TKI in which pro-atherothrombotic genetic status, traditional CV risk factors and biochemical profile, will be evaluated from baseline and monitored during treatment. The ultimate goal of this study is to provide a genetic and biochemical tool that will aid clinical evaluation in identifying patients with relevant atherothrombotic risk earlier and better, in order to offer them the most effective, yet safest, personalized TKI treatment.

## MATERIAL AND METHODS

### Patient population

Between May 2013 and October 2013, all consecutive patients with CP-CML referring to our out-patient clinic for a routine follow-up visit during imatinib or nilotinib treatment, with CCyR and with various degree of molecular response, were recruited for the study. Written informed consent was obtained in all subjects. Each participant was evaluated for: presence and onset-time of traditional CV risk factors (diabetes mellitus, dyslipidaemia, arterial hypertension, BMI, smoking habit, family history for vascular events); concurrent medications; presence of PAOD and other CV events occurring during TKIs treatment. An atherothrombotic event was taken into consideration if any of the following occurred: PAOD, acute cardiac syndrome, atherosclerotic cerebral ischemia and carotid atherosclerosis (stenosis > 50%). At the same time peripheral blood and serum samples were obtained to assess the following: intron 4 IVS4-14 A > G polymorphisms of OLR1 (rs3736235), distribution of genotypes AA (low risk), AG and GG (high risk); sCD40L, ox-LDL, IL-6, IL-10, TNF-α, hs-CRP, ETP, complete lipid profile (including LDL calculated by the Friedewald's formula) and fasting plasma glucose.

### Genetic analysis

Genomic DNA from peripheral blood leukocytes was extracted using the NucleoSpin Blood QuickPure Kit (MACHEREY-NAGEL). All samples were genotyped for the OLR1/Intron4 IVS4-14 A > G polymorphism by allelic discrimination assay using the TaqMan SNP Genotyping Assay (rs3736235; Applied Biosystem)

### Biochemical analysis

Plasma samples for biochemical analysis were harvested after centrifugation within two hours from blood collection and immediately stored at −80°C until processing.

#### Endogenous thrombin potential (ETP)

ETP was measured in platelet-poor plasma using a commercially available assay (Siemens, Marburg, Germany) in a BCS-XP System (Siemens, Marburg, Germany) according to manufacturer instructions. Coagulation activation was initiated by incubation of plasma with phospholipids, human recombinant tissue factor (Innovin; Siemens, Marburg, Germany), and calcium ions in the absence of thrombomodulin. The concentration of phospholipids and tissue factor is confidential to the manufacturer. Thrombin generation and subsequent inactivation was recorded by monitoring conversion of a specific slow reacting chromogenic substrate at a wavelength of 405 nm over time.

#### Soluble CD40 ligand

sCD40L was evaluated according to manufacturer instructions as previously reported and specific recommendations regarding the appropriate specimen and preparation for laboratory evaluation by Platinum ELISA kits from eBioscience, Ltd. (Hatfield, Ireland, United Kingdom) were used.

#### IL-6, TNF alpha, IL-10, ox-LDL

According to each manufacturer's specifications, IL-6 and TNF-α plasma levels were determined by using the high sensitivity Quantikine ELISA assays (R&D systems, Minneapolis, Minnesota); the Platinum ELISA kit from eBioscience, Ltd. (Hatfield, Ireland, United Kingdom) was employed for IL-10; oxLDL levels were determined by using ox-LDL Immundiagnostik AG - Bensheim, Germany ELISA Kit.

#### High-sensitivity C-reactive protein

Serum hs-CRP levels were measured using an available high sensitivity-CRP commercial kit (Siemens, CardioPhase, Marburg, Germany) based on an immunonephelometry assay.

#### ELISA equipment

Each ELISA test was performed on a Bio-Rad iMark Microplate Absorbance Reader (Biorad, Hercules, CA) except for sCD40, IL-10 and ox-LDL assays that were done on DSX automatized processor (DSX™, Bouty-Technogenetics Milan, Italy), provided by a specific software to perform the entire analytical process. All ELISA samples were assayed in duplicates.

### Statistical analysis

The Cox proportional-hazards modelling was used to evaluate the putative relations among end-points and the nonparametric data such as the genetic trait in the whole cohort of 110 subsequent CP CML patients in CCyR. For this purpose, the calculated number to detect a significant difference in parametric and not parametric variables, including genetic trait, evaluated, with 90% power at *p* = 0.01 was 96, if at least 14 events would occur. Accordingly, the statistical model consisting of a formal test for interaction was employed to determine the putative relation for each single variable. Final validation of data was assessed by a resampling technique (exact tests in SPSS 2003 module) and discrimination analysis by the Hosmer-Lemeshow method assuming a *p* < 0.05 as indicating a statistical significance. Furthermore, assuming a putative 10% difference in the frequency of events in association with each analyzed variable (single or cluster), according to previous data [42] and reported incidence and prevalence of PAOD in the general Caucasian population affected by traditional CV risk factors stratified for age [43], the calculated sample size to detect a significant difference in the genetic variables evaluated, with 90% power at *p* = 0.01, was 51 for each putative arm emerging from the whole cohort analysis.

To estimate putative differences in the levels of each single biochemical variable we employed the Mann Whitney U-test and the Wilcoxon test for comparisons between and within groups. The Kendall rank correlation coefficient was used to measure the relationship among measurable variables. Differences in genotype and allele proportion of the population and differences between observed and expected genotype frequencies (assuming Hardy-Weinberg equilibrium) were evaluated by χ^2^ test of independence. All calculations were performed using the SPSS library version 13 (SPSS Inc. Chicago, IL).

## SUPPLEMENTARY MATERIALS TABLES


