# Supplementary material for: Genetic predisposition and induced pro-inflammatory/pro-oxidative status may play a role in increased atherothrombotic events in nilotinib treated chronic myeloid leukemia patients
Source: Oncotarget. 2016 Aug 5;7(44):72311–21. doi: 10.18632/oncotarget.11100 (PMC5342164; doi:10.18632/oncotarget.11100)
Supplement: Supplementary file 1 [file oncotarget-07-72311-s001.pdf]

Genetic predisposition and induced pro-inflammatory/pro-oxidative status may play a role in increased atherothrombotic events in nilotinib treated chronic myeloid leukemia patients

Supplementary Material

Table S1: Clinical and biochemical parameters in the studied population according to TKI treatment and cardiovascular event

	**imatinib**			**nilotinib**			**whole cohort CV event free**	**whole cohort CV event**	**matched controls**
	all patients (n=65)	without tcrf # (n=29)	treated for tcrf # (n=19)	all patients (n= 45)	without tcrf # (n= 19)	treated for tcrf # (n= 24)	(n= 93)	(n= 17)	(n= 60)
**SAP** (mmHg)	140<br>(135-145)	138<br>(135-141)	142<br>(139-145)	138<br>(130-145)	136<br>(130-142)	140<br>(135-145)	138<br>(130-145)	140<br>(135-145)	135<br>(130-140)
**DAP** (mmHg)	85<br>(80-90)	85<br>(80-90)	88<br>(85-91)	86<br>(80-92)	85<br>(80-90)	88<br>(85-92)	86<br>(80-92)	89<br>(85-92)	85<br>(80-90)
**LDL** (mg/dl)	117<br>(109-125)	119<br>(111-127)	114<br>(105-123)	124<br>(118-130)	122<br>(118-126)	127<br>(121-133)	115<br>(109-130)	126<br>(121-133)	115<br>(100-130)
**HDL** (mg/dl)	50<br>(46-52)	48<br>(45-51)	48<br>(44-52)	49<br>(46-52)	50<br>(48-52)	48<br>(46-50)	50<br>(46-42)	48<br>(44-50)	50<br>(45-55)
**TG** (mg/dl)	118<br>(110-126)	114<br>(107-121)	122<br>(118-126)	120<br>(108-132)	116<br>(108-124)	124<br>(117-132)	118<br>(107-126)	123<br>(110-132)	120<br>(110-130)
**Glucose** (mg/dl)	94<br>(86-102)	96<br>(90-102)	92<br>(86-98)	97<br>(87-108)	94<br>(87-101)	100<br>(92-108)	94<br>(86-102)	98<br>(90-108)	95<br>(90-100)
**Hs-CRP** (mg/dl)	1.1<br>(0.8-1.4)	1.0<br>(0.8-1.2)	1.2<br>(1.0-1.4)	1.4<br>(1.1-1.7)	1.3<br>(1.1-1.5)	1.5<br>(1.3-1.7)	1.1<br>(0.8-1.4)	1.4<br>(1.1-1.7)	1<br>(0.7-1.3)
**Ox-LDL** (UI/L)	70<br>(62-78)	68<br>(62-74)	72<br>(66-78)	92<br>(82-103)*†	89<br>(82-96) *†	95<br>(87-103) *†	76<br>(62-84)	93<br>(87-103)§	65<br>(60-70)
**sCD40L** (pg/ml)	330<br>(271-388)	326<br>(271-380)	334<br>(280-388)	513<br>(434-601) *†	508<br>(434-582) *†	518<br>(435-601) *†	330<br>(271-390)	514<br>(388-601)§	350<br>(320-370)
**ETP** (%)	7<br>(6-8)	6.7<br>(6-7.4)	7.3<br>(6.6-8)	15<br>(11-19) *†	14<br>(11-17) *†	16<br>(13-19) *†	7<br>(6-7.4)	14<br>(11-19)§	5<br>(2.5 -7.5)
**TNFα** (pg/ml)	10<br>(8-12)	9.3<br>(8-10.6)	10.7<br>(9.4-12)	11<br>(9-13)†	12<br>(11-13)	10<br>(9-11)	10.1<br>(8-12.6)	11<br>(9.4-13)	9.5<br>(8-11)
**IL6** (pg/ml)	9<br>(7.5-10.5)	9.4<br>(8.3-10.5)	8.6<br>(7.5-9.7)	10<br>(8-12)	9.5<br>(8-11)	10.5<br>(9-12)	9.1<br>(7.5-10.5)	10.4<br>(8-12)	9<br>(8-10)
**IL10** (pg/ml)	4.9<br>(3.8-6)	5<br>(4-6)	4.8<br>(3.8-5.8)	1.1<br>(0.6-1.7)†	1<br>(0.6-1.4) *†	1.2<br>(0.7-1.7) *†	4.6<br>(1.6-6)	1.1<br>(0.6-1.7)§	5<br>(4-6)

Abbreviation: CV cardiovascular, TCRF traditional cardiovascular risk factors, SAP Systolic arterial pressure, DAP diastolic arterial pressure, LDL low-density lipoprotein cholesterol, HDL high-density lipoprotein cholesterol, TG triglycerides, hs-CRP high-sensibility C-reactive protein, ox-LDL oxidized-LDL, sCD40L soluble CD40 ligand, ETP endogenous thrombin potential, TNFα Tumor necrosis factor alpha, IL6 Interleukin-6, IL10 Interleukin-10.

To convert in mmol/L ° x 0.02586, °° x 0.01143

Mann-Whitney U test; * $p< 0.05$ between TKIs, † $p<0.05$ TKIs vs controls, § $p< 0.05$ patients affected by vascular events vs event free.

statins, and/or ace-inhibitors/angiotensin receptor blockers and/or oral glucose lowering drugs and/or antiplatelet drugs.

Table S2. **Correlations between antropometric and biochemical variables in CML patients treated with imatinib (A) or nilotinib (B). In each cell, the first value represents the Kendall rank correlation Coefficient, the second value (in Italics) the P val**

A:IMATINIB

Variable	TNF-α	IL-6	IL-10	Ox-LDL	sCD40L	ETP	Hs-CRP	LDL	HDL	TG	Glucose	SAP	DAP	BMI
**TNF-α**	**1**													
**IL-6**	0.195	**1**												
	0.058													
**IL-10**	0.195	0.075	**1**											
	-0.148	*0.273*												
**Ox-LDL**	0.172	0.133	-0.204	**1**										
	0.070	*0.142*	0.058											
**sCD40L**	0.186	**0.229**	-0.202	**0.233**	**1**									
	0.061	***0.046***	*0.059*	***0.041***										
**ETP**	0.147	0.128	-0.189	0.180	**0.246**	**1**								
	0.090	*0.099*	*0.085*	*0.074*	***0.030***									
**Hs-CRP**	0.116	**0.239**	-0.185	0.189	0.161	0.172	**1**							
	0.109	***0.045***	*0.077*	*0.080*	*0.090*	0.088								
**LDL**	0.188	0.132	-0.189	**0.241**	0.159	0.187	0.181	**1**						
	0.065	*0.099*	*0.079*	***0.041***	*0.101*	*0.061*	*0.072*							
**HDL**	-0.119	-0.051	0.197	-0.188	**-0.220**	-0.177	**-0.211**	0.177	**1**					
	0.370	*0.341*	*0.061*	*0.085*	***0.040***	*0.070*	***0.048***	*0.085*						
**TG**	0.194	0.077	-0.113	0.172	0.140	0.098	0.052	0.135	-0.199	**1**				
	0.061	*0.268*	*0.181*	*0.104*	*0.112*	*0.308*	*0.338*	*0.119*	*0.052*					
**Glucose**	0.199	0.141	-0.169	0.161	0.192	0.116	0.101	0.118	-0.175	0.182	**1**			
	0.059	*0.107*	*0.097*	*0.099*	*0.054*	*0.247*	*0.197*	*0.171*	*0.072*	*0.064*				
**SAP**	0.092	**0.232**	-0.090	0.067	0.104	0.157	0.144	0.099	-0.108	0.096	0.127	**1**		
	0.229	***0.046***	*0.235*	*0.295*	*0.163*	*0.096*	*0.094*	*0.213*	*0.102*	*0.128*	*0.103*			
**DAP**	-0.037	0.087	-0.144	0.183	0.153	0.128	0.107	0.118	-0.045	0.109	0.139	**0.254**	**1**	
	0.384	*0.241*	*0.123*	*0.071*	*0.106*	*0.152*	*0.196*	*0.171*	*0.359*	*0.102*	*0.108*	***0.038***		
**BMI**	0.029	0.147	-0.186	0.129	0.124	0.101	0.175	0.025	-0.183	**0.208**	0.194	0.119	0.158	**1**

	0.408	*0.094*	*0.080*	*0.118*	*0.127*	*0.208*	*0.076*	*0.420*	*0.069*	***0.046***	*0.058*	*0.090*	*0.077*	

B:NILOTINIB

Variable	TNF-α	IL-6	IL-10	Ox-LDL	sCD40L	ETP	Hs-CRP	LDL	HDL	TG	Glucose	SAP	DAP	BMI
**TNF-α**	1													
**IL-6**	0.198	**1**												
	0.054													
**IL-10**	- 0.178	-0.139	**1**											
	0.063	*0..099*												
**Ox-LDL**	0.118	0.153	**-0.454**	**1**										
	0.117	*0.092*	***<0.001***											
**sCD40L**	0.136	0.189	**-0.390**	**0.453**	**1**									
	0.094	0.069	***0.003***	***<0.001***										
**ETP**	0.107	0.128	**-0.388**	**0.445**	**0.446**	**1**								
	0.114	*0.110*	***0.003***	***0.001***	***0.001***									
**Hs-CRP**	0.196	0.169	-0.178	0.181	0.161	0.172	1							
	0.055	*0.070*	*0.083*	*0.081*	*0.099*	*0.090*								
**LDL**	0.189	0.122	-0.182	0.175	0.189	0.187	0.161	**1**						
	0.063	*0.108*	*0.081*	*0.088*	*0.079*	*0.066*	*0.084*							
**HDL**	-0.116	-0.091	0.159	-0.190	-0.190	-0.167	-0.193	0.157	**1**					
	0.114	*0.119*	*0.088*	*0.079*	*0.061*	*0.075*	*0.058*	*0.090*						
**TG**	0.154	0.097	-0.119	0.162	0.140	0.148	0.102	0.130	**-0.206**	**1**				
	0.081	*0.112*	*0.177*	*0.091*	*0.112*	*0.096*	*0.208*	*0.099*	***0.049***					
**Glucose**	0.180	0.122	-0.178	0.181	0.162	0.176	0.130	0.120	-0.169	0.164	**1**			
	0.072	*0.116*	*0.083*	*0.081*	*0.081*	*0.087*	*0.112*	*0.161*	*0.089*	*0.090*				
**SAP**	0.162	0.191	-0.096	0.097	0.114	0.148	0.144	0.102	-0.108	0.096	0.150	**1**		
	0.074	*0.060*	*0.221*	*0.166*	*0.153*	*0.097*	*0.100*	*0.208*	*0.186*	*0.184*	*0.099*			

**DAP**	0.097	0.099	-0.109	0.113	0.103	0.140	0.127	0.110	-0.050	0.099	0.130	**0.233**	**1**	
	0.154	*0.119*	*0.146*	*0.117*	*0.176*	*0.102*	*0.166*	*0.196*	*0.328*	*0.198*	*0.138*	***0.030***		
**BMI**	0.137	0.136	-0.166	0.149	0.114	0.111	0.167	0.045	-0.175	0.196	0.173	0.121	0.137	**1**
	0.101	*0.101*	*0.093*	*0.095*	*0.148*	*0.166*	*0..082*	*0.388*	*0.088*	*0.059*	*0.088*	*0.089*	*0.101*	

Abbreviation: SAP Systolic arterial pressure, DAP diastolic arterial pressure, LDL low-density lipoprotein cholesterol, HDL high-density lipoprotein cholesterol, TG triglycerides, hs-CRP high-sensibility C-reactive protein, ox-LDL oxidized-LDL, sCD40L soluble CD40 ligand, ETP endogenous thrombin potential, TNFα Tumor necrosis factor alpha, IL6 Interleukin-6, IL10 Interleukin-10, BMI body mass index.
